# Association of apolipoprotein A1 (rs 5069) genotyping with 25-hydroxyvitamin D deficiency and insulin resistance as a metabolic and genetic difference in obesity and type 2 diabetes mellitus

**DOI:** 10.1007/s00394-026-03938-4

**Published:** 2026-03-24

**Authors:** Nagla Usama, Amr E. Ahmed, Salma Mekheimer, Khaled Elhadidy, Mohamed Awadein, Mahmoud Farid

**Affiliations:** 1 Medical Laboratory Technology department, Faculty of Applied Health Science Technology, Misr University for Science and Technology, Cairo, Egypt; 2 Biotechnology and Life Sciences Department, Faculty of Postgraduate Studies for Advanced Sciences, Beni-Suef University, Beni-Suef, Egypt; 3 Internal Medicine Department, Faculty of Medicine, Beni-Suef University, Beni-Suef, Egypt; 4 Internal Medicine department, Faculty of Medicine, Misr University for Science and Technology, Cairo, Egypt

**Keywords:** 25(OH) vitamin D, Obesity, Type 2 diabetes mellitus, APOA1 rs5069, Genetic association

## Abstract

**Background:**

Obesity and type 2 diabetes mellitus (T2DM) are significant issues for public health, frequently occurring together with vitamin D deficiency and metabolic diseases. This study aimed to investigate whether the APOA1 single nucleotide polymorphism rs5069 is associated with 25-hydroxyvitamin D (25[OH]D) deficiency and insulin-resistance–related traits in Egyptian adults.

**Methods:**

In this cross-sectional study, 350 Egyptian adults (age 35–55 years) were grouped as: (1) obese non-diabetic (*n* = 100), (2) obese with T2DM (*n* = 100), (3) non-obese with T2DM (BMI < 30, *n* = 50), and (4) controls (BMI < 30 and 25[OH]D ≥ 20 ng/mL, *n* = 100). We measured BMI, 25(OH)D, FBS, HbA1c, fasting insulin, HOMA-IR and lipid profile. APOA1 rs5069 genotypes were determined by TaqMan assays. Group comparisons, Pearson correlation, and multivariate logistic regression (three models with incremental adjustments) were performed.

**Results:**

Vitamin D deficiency (25[OH]D < 20 ng/mL) was common in obese and diabetic groups (*p* < 0.001). Obese participants with T2DM had the worst metabolic profile (higher FBS, HbA1c, fasting insulin and HOMA-IR; dyslipidemia). The AA genotype and A allele of rs5069 were more frequent in obesity and T2DM than controls (genotype *p* = 0.005–0.011; allele *p* = 0.002–0.005). In obese groups, higher 25(OH)D levels were independently associated with lower odds of carrying GA and AA genotypes versus GG after adjustment for glycemic and lipid variables (models 2 and 3). No significant genotype–vitamin D association was observed in non-obese T2DM.

**Conclusion:**

APOA1 rs5069 (AA genotype) is associated with obesity and T2DM in this Egyptian cohort. Higher 25(OH)D was associated with reduced odds of GA/AA genotypes in obese participants, suggesting an interaction between vitamin D status and APOA1-related genetic susceptibility. Further longitudinal and mechanistic studies are needed.

## Introduction

Vitamin D deficiency, type 2 diabetes mellitus (T2DM), and obesity are interrelated global public health challenges that significantly contribute to metabolic and cardiovascular morbidity [1, 2]. Several studies have demonstrated that vitamin D deficiency is associated with obesity, impaired glycemic control, and an increased risk of diabetes complications [3, 4].

Although the underlying mechanisms are not fully understood, an inverse association between plasma 25-hydroxyvitamin D [25(OH)D] levels and body mass index (BMI) has been consistently observed [5]. One possible explanation is the volumetric dilution hypothesis, where vitamin D is sequestered and distributed in a larger fat mass, leading to lower circulating concentrations among obese individuals. Although vitamin D levels are similar in obese and lean individuals, its distribution in a larger fat volume leads to relatively lower concentrations in obese populations. Additionally, Weight loss has been shown to increase 25(OH)D levels, though the magnitude of this improvement varies among studies [6].

Vitamin D deficiency may also exacerbate adiposity through metabolic pathways. Reduced vitamin D levels can lower insulin secretion and increase lipogenesis, contributing to fat accumulation [7]. Moreover, vitamin D deficiency has been linked to decreased vitamin D receptor (VDR) expression in adipose tissue, impairing lipid mobilization and energy metabolism. In T2DM, low vitamin D levels are correlated with elevated HbA1c and insulin resistance, suggesting a regulatory role of vitamin D in glucose homeostasis and insulin sensitivity [8].

The Apolipoprotein A1 (APOA1) gene encodes the major structural protein of high-density lipoprotein (HDL), which is essential for reverse cholesterol transport, lipid binding, and activation of lecithin–cholesterol acyltransferase (LCAT). Genetic polymorphisms in APOA1 have been associated with variations in HDL-cholesterol levels, lipid metabolism, and susceptibility to metabolic disorders such as obesity, insulin resistance, and T2DM [9].

The APOA1 rs5069 polymorphism (a cytosine-to-thymine substitution at + 83 bp in the first intron) is a potentially functional variant [10]. Although located in a non-coding region, it may influence APOA1 gene expression by altering transcriptional regulation, enhancer activity, or RNA splicing. This variant has been associated with altered HDL-C concentrations and lipid metabolism in different populations, suggesting a role in modulating cardiovascular and metabolic risk [11]. This polymorphism was selected for analysis because of evidence linking rs5069 to variations in HDL levels, insulin resistance, and obesity risk. Experimental studies have also demonstrated that ApoA1 exerts anti-obesity effects, including stimulation of adipocyte lipolysis and reduction of adipose tissue mass. Conversely, ApoA1 deficiency in animal models leads to increased body weight and fat accumulation [12, 13]. This study aimed to investigate whether the APOA1 single nucleotide polymorphism rs5069 is associated with 25-hydroxyvitamin D (25[OH]D) deficiency and insulin-resistance–related traits in Egyptian adults.

## Materials and methods

A cross-sectional study was assessed 350 Egyptian cases, divided into 100 obese cases, consisting of (31% males, 69% females), their body mass index is more than 30. The 100 obese cases diagnosed with T2DM consisted of (38% males, and 62% females); 50 non-obese cases with T2DM (38% males, 62% females) their body mass index was less than 30; and their serum 25-hydroxyvitamin D level was less than 20 ng/dl. and 100 cases as controls consisted of (29% males, 71% females); their body mass index was less than 30. and their serum 25-hydroxyvitamin D level was more than 20 ng/dl.

Participants were recruited from the diabetic clinic at Souad Kafafi University Hospital over a defined period (e.g., January 2023 to December 2023). Eligible individuals were approached during their routine clinic visits and provided with detailed information about the study’s objectives, procedures, and potential risks and benefits. Those who expressed interest underwent a screening process to assess eligibility based on predefined inclusion and exclusion criteria. Each case provided written informed consent, and the MUST University Ethics Committee (FWA00025577) approved the research protocol.

Inclusion criteria were adults aged 35–55 years of both sexes, classified into four groups based on BMI and diabetic status. Diagnosis of T2DM was based on the American Diabetes Association (ADA) guidelines.

Exclusion criteria included individuals with a history of chronic liver or kidney disease, autoimmune or infectious diseases, recent use vitamin D supplementation, or medications influencing glucose or lipid metabolism, pregnancy or breastfeeding, smoking, and other factors potentially affecting vitamin D metabolism or metabolic status.

The study methodology involved assessment of baseline and clinical data, biochemical analysis, and genetic testing among the study group. The baseline and clinical data collected included age, sex, and BMI. Biochemical analyses focused on 25-hydroxyvitamin D, fasting blood sugar, HbA1c, fasting insulin, HOMA-IR, and lipid profile parameters. Blood samples were collected from all participants after an overnight fast using three different types of anticoagulant tubes: an EDTA-containing tube for DNA extraction and HbA1c measurement, a sodium fluoride-containing tube for fasting blood sugar, and a tube without anticoagulant for lipid profile and fasting insulin analysis. Fasting blood sugar and lipid profile were measured using an XL180 fully automatic clinical chemistry analyzer, with LDL cholesterol calculated via the Friedewald equation. HbA1c levels were determined using the turbidimetric method, and 25-hydroxyvitamin D levels were measured using the PerkinElmer ELISA kit (catalog number 10501), with deficiency criteria based on Trimboli et al. [14]. Insulin concentrations were assessed using the Chemux Bioscience ELISA kit (catalog number 1080), and the HOMA1-IR index was calculated to evaluate insulin resistance [15].

### Genetic analysis

Genomic DNA was extracted using the Thermo Scientific GeneJET Whole Blood Genomic DNA Purification Mini Kit. The APOA1 gene polymorphism (rs5069) was analyzed using the Applied Biosystems TaqMan SNP Genotyping Assay (Chr.11:116,837,538, Build GRCh38; [VIC/FAM] GAAGACCTCAGGTACCCAGAGGCCC[G/A] GCCTGGGGCAAGGCCTGAACCTTGA). The PCR was performed in a 10 μL reaction mixture containing TaqMan Master Mix, assay stock, and DNA template following standardized cycling conditions. The PCR process was conducted in a 10 μl reaction mixture, including TaqMan Master Mix, assay working stock, and DNA sample, following a standardized cycling protocol: initial denaturation at 95°C, followed by 40 cycles of denaturation and annealing/extension, with a final extension at 60 °C. This methodology ensured accurate detection and analysis of the targeted genetic polymorphisms and biochemical markers in the study group.

### Statistical analysis

Data were analyzed using SPSS version 26.0 (IBM Corp., Armonk, NY, USA). Continuous variables were expressed as mean ± standard deviation (SD), and categorical data as frequencies and percentages. Normality was tested using the Shapiro–Wilk test. Between-group comparisons were performed using one-way ANOVA with Duncan’s post hoc test for continuous variables, and chi-square test for categorical variables. Pearson correlation analysis was used for exploratory relationships between 25(OH)D and biochemical parameters. Multivariate logistic regression models were used to assess associations between 25(OH)D deficiency and APOA1 genotypes, adjusting sequentially for glycemic and lipid parameters. Odds ratios (ORs) and 95% confidence intervals (CIs) were calculated. ORs represent the odds of carrying GA or AA genotypes (vs. GG as reference) per unit increase in 25(OH)D (ng/mL). A two-tailed *p*-value < 0.05 was considered statistically significant.

## Results

The Baseline characteristics and biochemical characteristics of the study group summarize in Tables 1 and 2 as follows: The study group consisted of 350 individuals divided into four groups: 100 obese cases (31% males, 69% females), 100 obese cases with T2DM (44% males, 56% females), 50 non-obese cases with T2DM (38% males, 62% females), and 100 control cases (29% males, 71% females). The mean age ranged from 42.7 to 43.3 years across the groups. All studied groups showed no statistically significant difference with age or sex (*P*-value > 0.05). and had significant differences with BMI (*P*-value < 0.05).

**Table 1:** Baseline and clinical characteristics of study groups (N = 350)

Parameter	Mean (µ ± SD), n%				
	Control group N = 100	Obese cases N = 100	Obese cases with T2DM N = 100	T2DM their BMI < 30 N = 50	*p*-value
Sex	Male (29) 29% Female (71) 71%	Male (31) 31% Female (69) 69%	Male (38) 38% Female (62) 62%	Male (22) 44% Female (28) 56%	0.219
Age	42.7 ± 3.7^**a**^	43.2 ± 4.6^**a**^	42.8 ± 5.4^**a**^	43.3 ± 3.5^**a**^	0.780
BMI	24.6 ± 2.2^**a**^	35.6 ± 4.7^**b**^	32.85 ± 3.4^**c**^	24.7 ± 2.2^**a, d**^	< 0.001

Values are presented as mean ± standard deviation (SD) for continuous variables and as number (percentage) for categorical variables. BMI: body mass index. Different superscript letters (a, b, c, d) denote statistically significant differences between groups based on post hoc comparisons following one-way ANOVA (*p* < 0.05).

Bold typing refers to a significant difference for the p-value.

**Table 2:** Biochemical data analysis for all cases

Parameter	Mean (µ ± SD), n%				*P*-value				
	Control group N = 100	Obese non- T2DM N = 100	Obese T2DM N = 100	Non-obese T2DM N = 50	Control Vs Obese non- T2DM	Control Vs Obese T2DM	Control Vs Non-obese T2DM	Obese non- T2DM Vs Obese T2DM	Obese T2DM Vs Non-obese T2DM
25(OH) vitamin D	25.2 ± 3.1	10.1 ± 4.9	10.2 ± 4.3	11.28 ± 5.04	< 0.001	< 0.001	< 0.001	0.868	0.191
FBS (mg/dl)	85.7 ± 9.3	105.6 ± 20.2	131.3 ± 40.6	103.15 ± 23.2	< 0.001	< 0.001	< 0.001	< 0.001	< 0.001
HbA1C %	4.7 ± 0.42	5.5 ± 0.7	7.8 ± 1.2	7.4 ± 0.91	< 0.001	< 0.001	< 0.001	< 0.001	0.027
Fasting insulin mU/L	5.4 ± 3.4	7 ± 4.8	8.2 ± 3.6	7.8 ± 4.3	0.007	< 0.001	< 0.001	0.046	0.523
HOMA-IR	1.1 ± 0.7	1.8 ± 1.3	2.6 ± 1.74	1.93 ± 1.01	< 0.001	< 0.001	< 0.001	0.002	0.002
TC (mg/dl)	152.5 ± 28.2	183.8 ± 38.5	181.2 ± 40.1	155.9 ± 29.65	< 0.001	< 0.001	0.497	0.632	< 0.001
TG (mg/dl)	129 ± 33.7	164.1 ± 62.7	148.7 ± 51	106.9 ± 32.07	< 0.001	0.002	< 0.001	0.059	< 0.001
HDL-C (mg/dl)	55.9 ± 10.2	55.5 ± 8.6	50.1 ± 9.1	55.1 ± 8.4	0.757	< 0.001	0.635	< 0.001	0.002
LDL-C (mg/dl)	70.0 ± 25.3	95.5 ± 33	101.3 ± 37.6	79.4 ± 30.1	< 0.001	< 0.001	0.091	0.248	< 0.001
TG/HDL-C ratio	1.96 ± 0.721	3.05 ± 1.3	3.1 ± 1.3	2.02 ± 0.83	< 0.001	< 0.001	0.009	0.729	< 0.001

Values are expressed as mean ± standard deviation (SD). FBS: fasting blood sugar; HbA1c: glycated hemoglobin; HOMA-IR: homeostatic model assessment of insulin resistance; TC: total cholesterol; TG: triglycerides; HDL-C: high-density lipoprotein cholesterol; LDL-C: low-density lipoprotein cholesterol.

Comparisons between groups were performed using one-way ANOVA followed by post hoc tests (Tukey) for pairwise differences. A *p*-value < 0.05 was considered statistically significant.

The prevalence of vitamin D deficiency (defined as serum 25(OH) vitamin D levels < 20 ng/mL) [14] was significantly higher in all diabetic and obese study groups compared to the control group (*p* < 0.001). There was no significant difference between the obese non-diabetic and obese T2DM groups (*p* = 0.868), nor between the obese T2DM and non-obese T2DM groups (*p* = 0.191). These findings suggest that vitamin D deficiency is prevalent among all diabetic and obese study groups, regardless of diabetes status or obesity.

Notable metabolic abnormalities were detected among the groups, with the obese diabetic group exhibiting the most significant abnormalities. The diabetic groups, especially obese diabetics, had significantly elevated levels of FBS, HbA1C, and fasting insulin, which suggests a strong resistance to insulin and insufficient control of blood sugar levels. The HOMA-IR index provided additional evidence of increased insulin resistance in these individuals. The lipid profiles indicated that the obese groups had higher levels of total cholesterol and triglycerides. Among these groups, the obese diabetic group had the highest levels of LDL-C and a lower HDL-C, The TG/HDL-C ratio was notably increased in all groups, particularly among individuals with diabetes.

Table 1. Baseline characteristics of the study groups. Sex distribution is shown as the percentage of males and females in each group. Age and BMI are presented as mean ± standard deviation (SD). p-values indicate statistical differences between the groups.

Table 2 Biochemical characteristics of the study groups. The table displays the mean values (± SD) of biochemical parameters and, including 25(OH) vitamin D, FBS, HbA1c, fasting insulin, HOMA-IR, TC, TG, HDL-C, LDL-C, and the TG/HDL-C ratio among the four study groups. *P*-values indicate the significance of differences between the groups for each parameter using t-test.

Pearson correlation analysis between 25(OH) vitamin D levels and biochemical parameters among all cases is presented in Table 3. The analysis demonstrated statistically significant correlations, where lower levels of 25(OH) vitamin D were correlated with higher fasting insulin levels in both obese non-diabetic and diabetic groups. A negative correlation between cholesterol levels and vitamin D was observed in the obese diabetic group. Notably, a contrasting relationship was found with triglycerides: in the control group, higher triglycerides were associated with higher vitamin D levels, whereas in the obese diabetic group, the correlation was negative. HDL-C and LDL-C did not show significant correlations with vitamin D across the groups. However, in the control group, a higher TG/HDL-C ratio correlated with higher vitamin D levels.

**Table 3:** Pearson correlation between 25(OH) vitamin D and biochemical data among all cases

25(OH) vitamin D								
parameters	Control		Obese non-T2DM		obese T2DM		Non-obese T2DM	
	r	*p*-value	r	*p*− value	r	*p*-value	r	*p*-value
BMI	− 0.097	0.335	− 0.117	0.248	0.236^*^	0.018	0.229	0.110
FBS	− 0.181	0.071	− 0.178	0.077	− 0.201^*^	0.045	0.127	0.378
HbA1C	− 0.058	0.569	0.002	0.982	0.036	0.722	− 0.009	0.952
F insulin	0.055	0.587	− 0.234^*^	0.019	− 0.201^*^	0.045	0.203	0.157
HOMA-IR	0.017	0.866	− 0.275	0.006	− 0.272	0.006	0.16	0.172
TC	− 0.042	0.676	0.041	0.686	− 0.230^*^	0.022	0.152	0.293
TG	0.374^**^	0.000	− 0.166	0.099	− 0.202^*^	0.043	0.156	0.280
HDL	0.104	0.303	− 0.053	0.599	− 0.093	0.356	− 0.115	0.427
LDL	− 0.166	0.098	0.125	0.216	− 0.167	0.096	0.148	0.305
TG/HDL ratio	0.203^*^	0.043	− 0.101	0.317	− 0.112	0.267	0.172	0.232

Table 3 Pearson correlation between 25(OH) vitamin D levels and biochemical parameters among study groups. The table displays the correlation coefficients (r) and *p*-values for the association between 25(OH) vitamin D levels and various biochemical markers, including BMI, FBS, HbA1c, fasting insulin, HOMA-IR, TC, TG, HDL-C, LDL-C, and TG/HDL-C ratio. Correlations are calculated separately for each study group. * p-value > 0.05, ** p-value > 0.001 

### The distribution of APOA1 genotypes and alleles frequencies in three studied groups illustrated in Table 4

**Table 4:** Distribution of APOE genotypes in all studied cases

APOA1 genotypes & alleles rs5069	Control group N = 100	Obese non- T2DM N = 100	Obese T2DM N = 100	Non-obese T2DM N = 50	*P*- value			
					Control Vs Obese non- T2DM	Control Vs Obese T2DM	Obese non- T2DM Vs Obese T2DM	Obese T2DM Vs Non-obese T2DM
GG (ref)	65 (65%)	40 (40%)	48 (48%)	25 (50%)	< 0.001	0.011	< 0.001	0.260
GA	35 (35%)	49 (49%)	37 (37%)	22 (44%)				
AA	0	11 (11%)	15 (15%)	3 (6%)				
G (ref)	165 (82.5%)	129 (64.5%)	133 (66.5%)	72 (72%)	< 0.001	0.005	0.674	0.334
A	35 (17.5%)	71 (35.5%)	67 (33.5%)	28 (28%)				

The Hardy–Weinberg equilibrium (HWE) test was applied to compare observed versus expected genotype frequencies for the APOA1 rs5069 single nucleotide polymorphism (SNP) across the four study groups. The results indicated statistically significant differences in the distribution of rs5069 genotypes among the groups.

Specifically, significant differences in APOA1 rs5069 genotypes and allele frequencies were observed between the control group and T2DM cases (*p* = 0.011 for genotypes, *p* = 0.005 for alleles). Similarly, significant differences were found between the control group and obese cases (*p* = 0.005 for genotypes, *p* = 0.002 for alleles). Furthermore, a significant difference in genotype distribution was detected between non-diabetic obese cases and obese T2DM cases (*p* < 0.001), whereas allele frequency differences were not significant (*p* = 0.674).

By studying the distribution of the Apolipoprotein A1 gene SNP (rs 5069) among obese T2DM patients and non-obese T2DM, the results revealed that there was no significant difference in distribution in (rs 5069) genotypes.

The associations of APOA1 gene polymorphisms and the risk of obesity and T2DM compared to the control group using univariate analysis illustrated Table 5. The odds ratios (ORs) presented in this table are crude ORs estimating the risk of obesity and T2DM associated with APOA1 rs5069 genotypes and alleles compared to the reference genotypes (GG for genotypes and G for alleles). The GA and AA genotypes and the A allele were evaluated for their contribution as potential risk factors in the development of obesity and T2DM**.** GG is the most frequent genotype between T2DM cases (48%) and control group (64%). AA was the most frequent genotype associated with T2DM cases (15%) OR = 0.762, 95% CI = 0.664–0.875, *P*-value = 0.003. and obese cases (11%) OR = 0.784 95% CI = 0.679–0.906, *P*-value = 0.005. While it is completely absence in the control group. A allele was a more frequent allele associated with T2DM (33.5%) cases OR = 0.436, 95% CI = 0.242–0.785, *P*-value = 0.005, and obese cases (35.5%) OR = 1.972, 95% CI = 1.247–3.118, *P*-value = 0.002 than in the controls group.

**Table 5:** Associations of APOA1 rs5069 genotypes and alleles as risk factors for obesity and type 2 diabetes mellitus (T2DM)

APOA1 genotypes & alleles rs5069	Control Vs Obese non- T2DM		Control Vs Obese T2DM		Obese non- T2DM Vs Obese T2DM		Obese T2DM Vs Non-obese T2DM	
	OR (95%CI)	*p*-value	OR (95%CI)	*p*-value	OR (95%CI)	*p*-value	OR (95%CI)	*p*-value
GG (ref)	–	–	–	–	–	–	–	–
GA	0.730 (0.35–1.49)	0.390	1.573 (1.13–2.18)	0.006	0.629 (0.34 -1.14)	0.128	0.876 (0.42–1.79)	0.717
AA	0.762 (0.66–0.87)	0.003	0.784 (0.67–0.90)	< 0.001	1.136 (0.46 -2.75)	0.777	2.604 (0.68–9.85)	0.148
G (ref)	–	–	–	–	–	–	–	–
A	0.436 (0.24–0.78)	0.005	1.972 (1.24–3.11)	0.002	2.029 (1.42–2.89)	< 0.001	1.295 (0.76–2.19)	0.334

Table 4 Distribution of APOA1 (rs5069) genotypes and alleles in the study groups. This table displays the genotype (GG, GA, AA) and allele (G, A) frequencies of the APOA1 rs5069 polymorphism among the four study groups. *p*-values indicate the statistical differences in genotype and allele distribution between the groups.

Table 5 Associations of APOA1 rs5069 genotypes and alleles as risk factors for obesity and T2DM. The table displays the odds ratios (OR) and 95% confidence intervals (CI) for the association of APOA1 rs5069 genotypes (GA, AA) and alleles (A) with the risk of obesity and T2DM, compared to the reference genotype (GG) and allele (G). *p*-values indicate the statistical significance of the associations

OR odds ratio; CI, confidence interval; ref, reference group; T2DM, type 2 diabetes mellitus. Statistical significance: p < 0.05 was considered statistically significant.

To evaluate the independent association of 25(OH) vitamin D deficiency with APOA1 genotypes, multivariate logistic regression analyses were conducted among obese cases (Table 6), obese cases with T2DM (Table 7), and non-obese T2DM cases (Table 8).

**Table 6:** Logistic regression analysis for APOA1 genotypes and 25 (OH) vitamin D among non-diabetic obese cases

	GA		AA	
	B	OR 95%CI	B	OR 95%CI
Model 1	− 0.26	0.77 (0.68–0.87) ***p*-value < 0.001**	− 0.48	0.61 (0.49–0.77) ***p*-value < 0.001**
Model 2	− 0.32	0.72 (0.62–0.84) ***p*-value > 0.001**	− 0.45	0.63 (0.49–0.805) ***p*-value > 0.001**
Model 3	− 0.32	0.724 (0.621–0.84) ***p*-value > 0.001**	− 0.53	0.58 (0.43–0.79) ***p*-value > 0.001**

Bold typing refers to a significant difference for the p-value.

**Table 7:** Logistic regression analysis for APOA1 genotypes and 25 (OH) vitamin D among obese cases with T2DM

	GA		AA	
	B	OR 95%CI	B	OR 95%CI
Model 1	− 0.246	0.78 (0.674–0.91) ***p*-value = 0.001**	− 0.509	0.60 (0.47–0.76) ***p*-value < 0.001**
Model 2	− 0.234	0.79 (0.67–0.92) ***p*-value = 0.003**	− 0.617	0.54 (0.40–0.72) ***p*-value > 0.001**
Model 3	− 0.248	0.78 (0.65 0.92) ***p*-value > 0.001**	− 0.662	0.516 (0.375–0.709) ***p*-value > 0.001**

Bold typing refers to a significant difference for the p-value.

**Table 8:** Logistic regression analysis for APOA1 genotypes and 25 (OH) vitamin D among non—obese T2DM

	GA		AA	
	B	OR 95%CI	B	OR 95%CI
Model 1	0.005	1.005 (0.89–1.12) *p*-value = 0.93	− 0.146	0.865 (0.64–1.16) *p*-value = 0.334
Model 2	− 0.022	0.97 (0.84–1.12) *p*-value = 0.75	− 0.199	0.820 (0.59–1.13) *p*-value = 0.227
Model 3	0.018	1.018 (0.86–1.19) *p*-value = 0.82	− 0.082	0.921 (0.60–1.403) *p*-value = 0.702

Three models were used:

Model 1: Unadjusted analysis.

Model 2: Adjusted for HbA1c, fasting insulin, and HOMA-IR.

Model 3: Further adjusted for lipid profile parameters in addition to Model 2 adjustments.

**Fig. 1:**
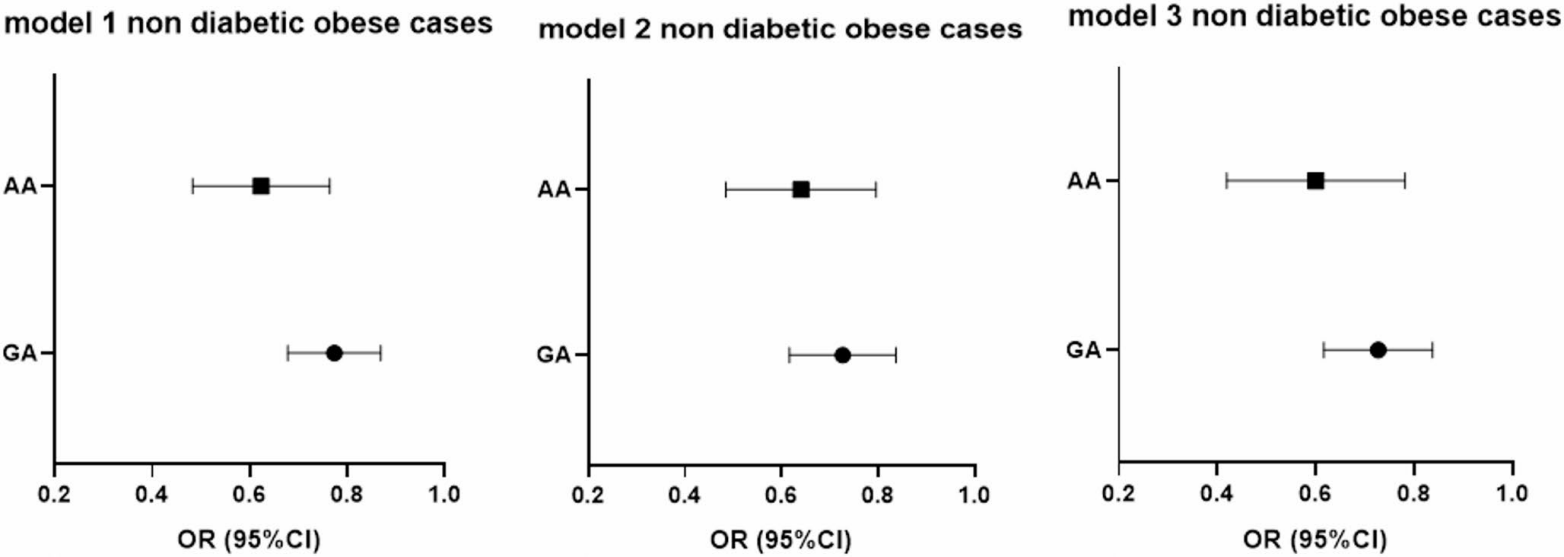
forest blot illustrates the OR (95%CI) for 25 (OH) vitamin D with APOA genotypes among non-diabetic obese cases

**Fig. 2:**
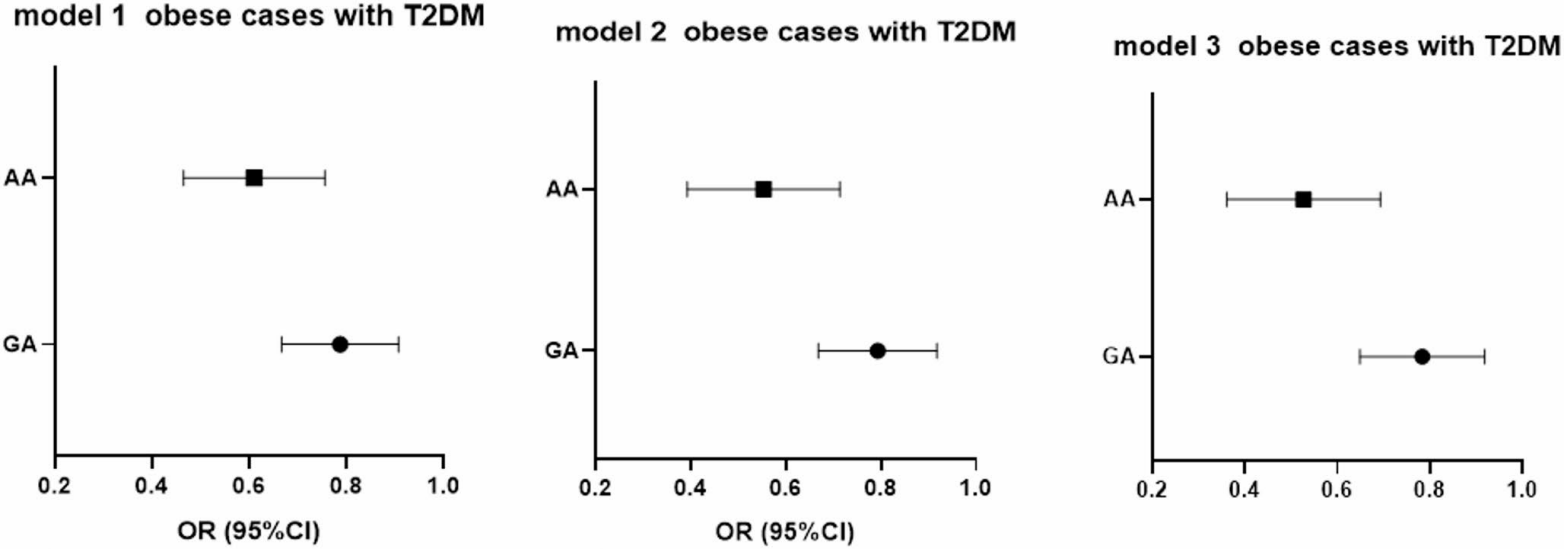
forest blot illustrates the OR (95%CI) for 25 (OH) vitamin D with APOA genotypes among non-diabetic obese cases

**Fig. 3:**
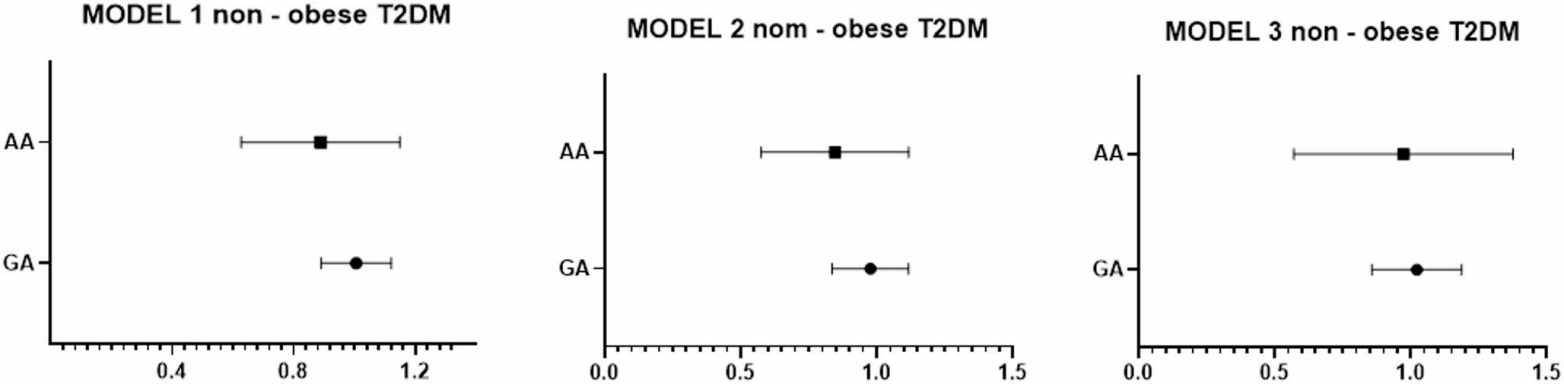
forest blot illustrates the OR (95%CI) for 25 (OH) vitamin D with APOA genotypes among non- obese T2DM cases

These models are illustrated in Figs. 1, 2 and 3.

The analysis demonstrated that, within the non-diabetic obese study group, higher serum vitamin D levels were associated with a significantly lower odds of carrying the GA and AA genotypes compared to the GG genotype. Specifically, the odds of having the GA and AA genotypes decreased by factors of approximately 0.26 and 0.48, respectively. These associations remained statistically significant (*p* < 0.05) even after adjusting for confounding variables in Models 2 and 3.

Among obese individuals with T2DM, higher vitamin D levels were likewise linked to decreased odds of GA and AA genotypes (*p* < 0.001), and this association persisted across adjusted models. This suggests a potential modulatory role of vitamin D in lipid-related gene expression among metabolically impaired obese individuals.

Conversely, in non-obese T2DM participants, no significant association was observed between vitamin D levels and APOA1 genotypes after adjustment, implying that the genotype–vitamin D relationship may be specific to obesity-related metabolic conditions.

These findings collectively indicate that vitamin D deficiency is associated with a greater likelihood of possessing the APOA1 variant genotypes among obese and diabetic individuals, highlighting an interaction between vitamin D metabolism and genetic susceptibility in metabolic disorders.

## Discussion

### Pathophysiological mechanisms in obesity and T2DM

Obesity is characterized by an increased accumulation of adipose tissue, leading to altered adipokine secretion and chronic inflammation. Adipose tissue, particularly visceral fat, secretes pro-inflammatory cytokines such as TNF-α, IL-6, and leptin while decreasing adiponectin levels. These changes disrupt insulin signaling, promoting insulin resistance and hyperglycemia. Obesity-related insulin resistance is also driven by lipotoxicity and ectopic fat deposition, primarily in the liver and skeletal muscles [16].

T2DM is marked by both insulin resistance and impaired insulin secretion. Chronic hyperglycemia resulting from insulin resistance leads to β-cell dysfunction and eventual pancreatic exhaustion. Persistent hyperglycemia also induces glycation of proteins and lipids, contributing to microvascular and macrovascular complications. In individuals with combined obesity and T2DM, the pathophysiological processes are amplified, leading to a more severe metabolic profile [17].

Vitamin D plays a crucial role in glucose metabolism through multiple mechanisms. It enhances insulin production by activating the vitamin D receptor (VDR) in pancreatic β-cells, a process mediated by the regulation of calcium influx essential for insulin exocytosis. Additionally, calcitriol increases insulin sensitivity by regulating insulin receptor expression, thereby promoting glucose uptake in skeletal muscle and adipose tissue. Vitamin D also exerts anti-inflammatory effects by inhibiting nuclear factor-kappa B (NF-κB) activation, which reduces the expression of pro-inflammatory cytokines and helps mitigate insulin resistance. Moreover, it modulates lipid metabolism by influencing lipogenesis and lipid oxidation, potentially decreasing ectopic fat accumulation [18].

Apolipoprotein A-I (APOA1), the main protein component of HDL, plays a crucial role in reverse cholesterol transport (RCT) and anti-inflammatory functions. The APOA1 (rs5069) polymorphism appears to influence HDL function and cholesterol efflux, which may contribute to altered lipid profiles in obese and diabetic individuals [19].

The study was conducted to investigate the association between APOA1 (rs5069) genotypes and 25(OH) vitamin D deficiency in relation to obesity and type 2 diabetes mellitus (T2DM). The results reveal significant metabolic and genetic differences among the four groups: obese individuals, obese individuals with T2DM, non-obese individuals with T2DM, and control cases. While age and sex showed no significant differences across groups, BMI was notably higher in the obese and obese diabetic groups compared to controls, reflecting the study's focus on obesity and diabetes. Vitamin D deficiency was prevalent across all diabetic and obese groups, with no significant differences between diabetic and non-diabetic obese individuals, or between obese and non-obese diabetic individuals.

Metabolic abnormalities were particularly pronounced in the obese diabetic group, as evidenced by elevated levels of fasting blood sugar (FBS), HbA1c, and fasting insulin, along with a higher HOMA-IR index, indicating substantial insulin resistance and poor glycemic control. The lipid profiles of the obese groups also revealed significant dyslipidemia, with elevated total cholesterol, triglycerides, and LDL-C levels, alongside reduced HDL-C levels, particularly in the obese diabetic group. These findings highlight the compounded metabolic risks in obese individuals with diabetes.

A significant negative correlation between low 25-hydroxyvitamin D levels and FBS, fasting insulin level, HOMA-IR, TC, and TG among obese T2DM cases, and a significant negative correlation between low 25-hydroxyvitamin D levels fasting insulin level as well as HOMA-IR among nondiabetic obese cases were conducted.

Previous studies showed a correlation between vitamin D deficiency and an undesirable lipid profile. It was shown to be associated with higher LDL-C, TG, [20] and Apo B [21] levels, while increased levels of good lipids, including HDL-C [22] and Apo A-1 have been linked to higher vitamin D levels [23]. A correlation study was conducted on 25(OH) vitamin D levels with lipids and showed that 25(OH) vitamin D levels are inversely correlated with TC, LDL-C, TG, non-HDL-C, LDL-P, sLDL-P, sdLDL-C, ApoB, and ApoB/A ratios and positively correlated with HDL2-C. Vitamin D deficiency increases the risk of dyslipidemia and coronary heart disease [24]. The mechanism behind the link between vitamin D and lipids is still unclear. Cholecalciferol is made by irradiating cutaneous 7-dehydrocholesterol, which is a precursor to cholesterol. Vitamin D is structurally linked to cholesterol [25].

In recent years, it has been discovered that vitamin D deficiency and T2DM are related [26]. vitamin D has important role in improving glycemia and insulin secretion because it stimulates the pancreas to produce insulin [27]. In addition, Lower vitamin D concentrations have been linked to increased BMI and dyslipidemia based on a study conducted by Auwerx et al. [28], serum vitamin D had a substantial positive relationship with both HDL-C and APOAI.

The genetic analysis focusing on APOA1 gene polymorphisms (rs5069) revealed significant differences in genotype and allele distributions between control groups and those with T2DM or obesity. The AA genotype was significantly associated with T2DM and obesity, suggesting a potential genetic predisposition. However, no significant differences in rs5069 genotype distribution were observed between obese and non-obese T2DM patients, indicating that this polymorphism may not distinguish between these two diabetic phenotypes.

Multivariate logistic regression analysis further demonstrated that higher levels of 25(OH) vitamin D were associated with reduced odds of carrying the GA and AA genotypes compared to the GG genotype in obese and obese diabetic individuals. This association remained significant even after adjusting for variables such as HbA1c, fasting insulin, HOMA-IR, and lipid profile parameters, suggesting that the protective effect of vitamin D on APOA1 genotyping is independent of its influence on lipid metabolism. However, in non-obese T2DM individuals, no significant association was found between 25(OH) vitamin D levels and APOA1 genotypes, indicating a complex interaction between genetic factors, vitamin D levels, and metabolic status.

In a Korean cohort study, APOA1 connected positively with vitamin D and genes involved with APOA1 and 25-hydroxyvitamin D [29]. Numerous recent studies have assessed the correlation between apolipoprotein SNPs and obesity, lipid profiles, and dyslipidemia [30, 31]. As well as the association of 25-hydroxyvitamin D deficiency with obesity and lipid profiles [32–34].

Recently, the processes by which apolipoproteins and HDL impact insulin action, a crucial feature of T2DM pathophysiology, have been investigated [35, 36]. The link between low HDL and the development of T2DM has previously been observed, with causation partially assessed as plasma insulin increased and plasma glucose reduced following an infusion of HDL (including apoA1) in T2DM patients [37]. Apolipoprotein A1 the principal HDL protein, is generated mostly in the small intestine and liver. APOA1/HDL particles carry excess cholesterol from peripheral tissues to the liver via the reverse cholesterol transport route [38]. Low blood 25(OH) D is related with low HDL-cholesterol, high serum total cholesterol (TC), LDL-cholesterol, and Apo B/Apo A1 ratio [39]. Numerous research have investigated the impact of vitamin D supplementation on Apo A1 levels [40–42]. A meta-analysis study summarized and quantified the effect of vitamin D intake on apolipoprotein A1 and B100 levels in adults to provide an evidence-based reference for therapeutic approaches.

The meta-analysis revealed that vitamin D intake had no significant effect on apolipoproteins A1 and B100 levels; however, Apo A1 levels increased significantly with daily vitamin D dosage and supplementation for 12 weeks [43]. On the other hand, we investigated the relationship between APOA1 (rs5069) genotyping and vitamin D deficiency and found that AA genotype and A allele were the most common genotype and allele linked with T2DM cases and vitamin D deficit compared to GG genotypes. In a genome-wide association study meta-analysis of two Korean cohorts encompassing 12,924 patients to find novel single nucleotide polymorphisms (SNPs) linked with ApoA1, genes connected with ApoA1, and vitamin D were identified [44].

A.A. Ibrahim.et al., revealed that TT and CT genotypes and T allele of APOA1 C + 83 T were significantly higher in T2DM with CAD cases than control [30].

According to a study by *FA Casillas. *et al., T2DM patients with myocardial infarction who carried the APOA1 polymorphism 83 C > T had lower HDL-C and triglyceride levels than those who carried the C/C genotype [45].

This study provides novel evidence linking the APOA1 rs5069 polymorphism with vitamin D deficiency, obesity, and T2DM in an Egyptian cohort, highlighting a potential gene–vitamin D interaction influencing metabolic risk. By integrating genetic, biochemical, and insulin-resistance parameters, the work offers a comprehensive perspective that extends beyond previous studies focused on isolated metabolic traits. Nevertheless, several limitations should be acknowledged. The cross-sectional design limits causal inference, and only a single polymorphism (rs5069) was analyzed without functional validation of APOA1 expression. Additionally, factors such as dietary vitamin D intake, sunlight exposure, and physical activity were not evaluated. The relatively small size of some subgroups and single-center recruitment may also restrict the generalizability of the findings. Future multicenter and longitudinal studies incorporating broader genetic profiling and functional assays are warranted to confirm and expand these results.

## Conclusion

This study highlights the significant metabolic and genetic differences among obese individuals, both with and without T2DM, non-obese individuals with T2DM, and healthy controls. Our findings underscore the pervasive issue of vitamin D deficiency across all diabetic and obese groups, which correlates negatively with several key metabolic parameters, including FBS, fasting insulin, and lipid profiles. The pronounced metabolic abnormalities observed in the obese diabetic group further emphasize the compounded risks associated with obesity and diabetes.

The genetic analysis revealed that the AA genotype of the APOA1 rs5069 polymorphism is significantly associated with obesity and T2DM, suggesting a genetic predisposition in these populations. Interestingly, higher levels of 25(OH) vitamin D were associated with a reduced likelihood of carrying the GA and AA genotypes, particularly in obese individuals, indicating a protective role of vitamin D that is independent of its influence on lipid metabolism.

## Data Availability

All data generated or analyzed during this study are included in this article.
